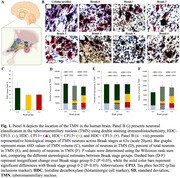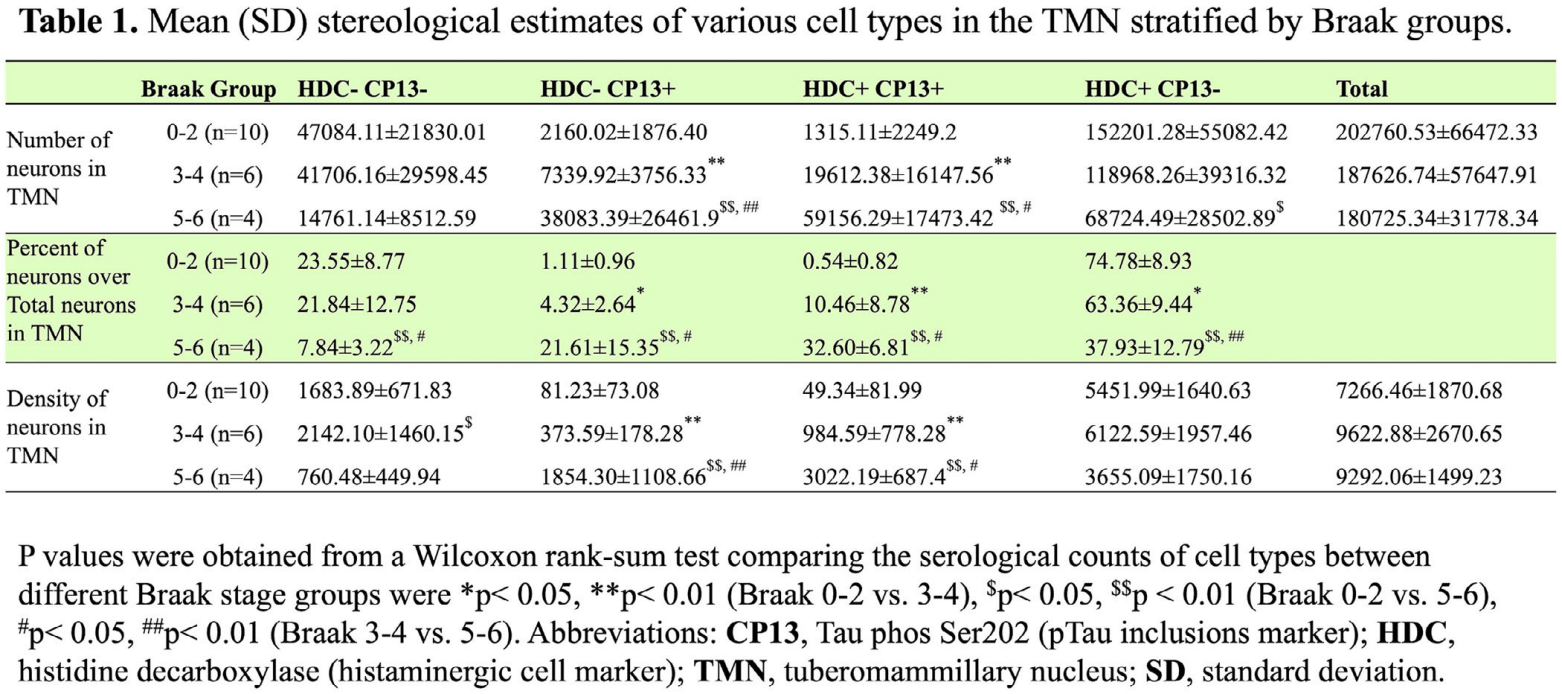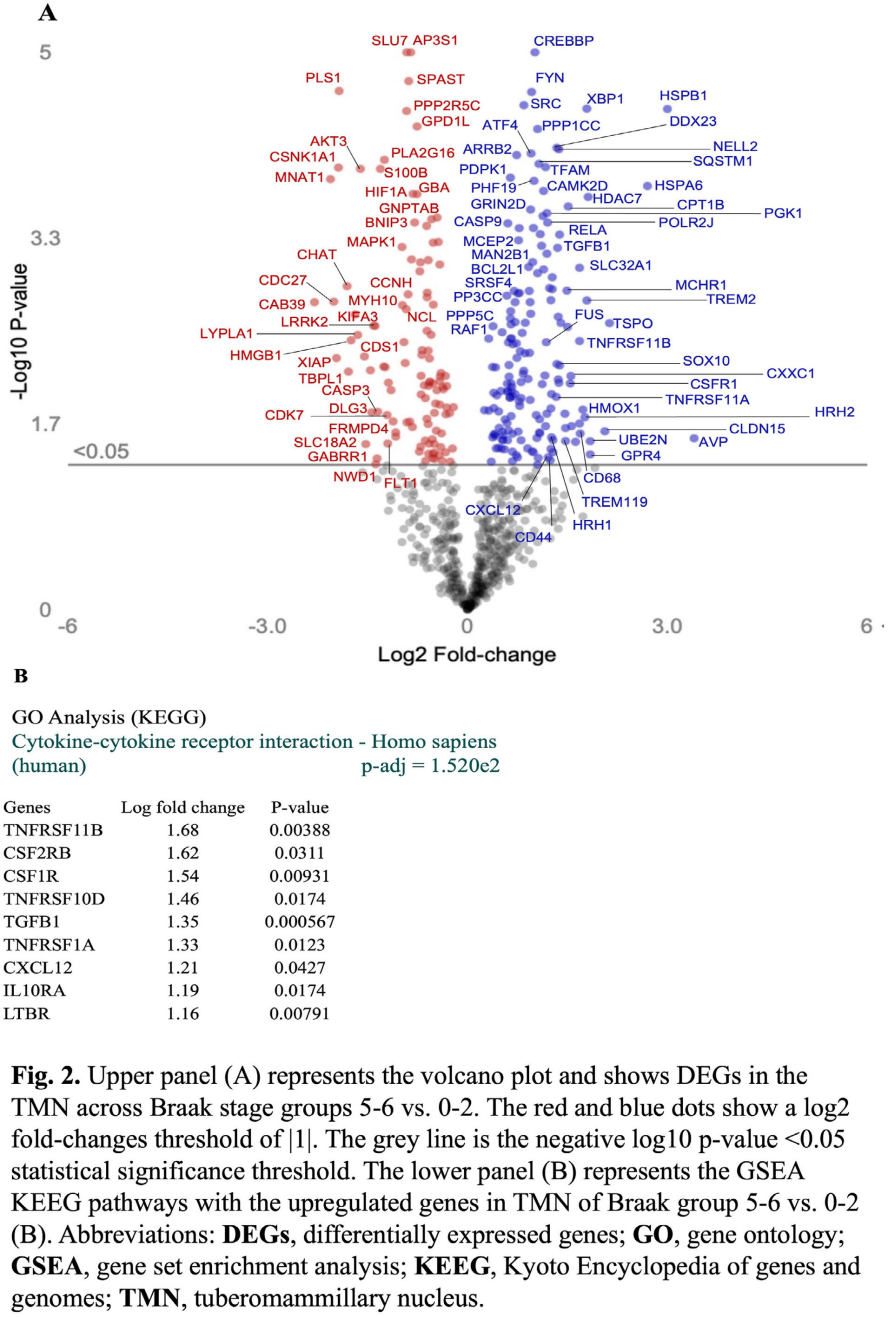# Preservation amidst decline: wake‐promoting histaminergic system provides a novel target to mitigate sleep/wake dysfunction in Alzheimer’s disease

**DOI:** 10.1002/alz.091638

**Published:** 2025-01-03

**Authors:** Abhijit Satpati, Felipe Luiz Pereira, Alexander V. Soloviev, Mihovil Mladinov, Renata Elaine Paraizo Leite, Claudia Kimie Suemoto, Roberta Diehl Rodriguez, Vitor Ribeiro Paes, Christine M Walsh, Salvatore Spina, William W. Seeley, Carlos Augusto Pasqualucci, Wilson Jacob‐Filho, Thomas C. Neylan, Lea T. Grinberg

**Affiliations:** ^1^ Memory and Aging Center, UCSF Weill Institute for Neurosciences, University of California San Francisco, San Francisco, CA USA; ^2^ Klinik und Poliklinik für Psychiatrie und Psychotherapie Universitätsmedizin Rostock, Rostock, Mecklenburg‐Vorpommern Germany; ^3^ Physiopathology in Aging Laboratory (LIM‐22), University of São Paulo Medical School, São Paulo, São Paulo Brazil; ^4^ Department of Pathology, University of Sao Paulo Medical School, São Paulo, São Paulo Brazil; ^5^ Physiopathology in Aging Laboratory (LIM‐22), Department of Internal Medicine, University of Sao Paulo Medical School, São Paulo, São Paulo Brazil; ^6^ Division of Geriatrics, University of São Paulo Medical School, São Paulo Brazil; ^7^ University of São Paulo Medical School, São Paulo, São Paulo Brazil; ^8^ Department of Psychiatry and Behavioral Sciences, University of California San Francisco, San Francisco, CA USA; ^9^ Memory & Aging Center, Department of Neurology, University of California in San Francisco, San Francisco, CA USA; ^10^ Global Brain Health Institute, University of California San Francisco, San Francisco, CA USA

## Abstract

**Background:**

Excessive daytime sleepiness is a common and early symptom of Alzheimer’s disease (AD). The subcortical wake‐promoting neurons in the lateral hypothalamic area, tuberomammillary nucleus (TMN), and locus coeruleus synchronize to maintain wakefulness/arousal. Although significant neuronal decline occurs in wake‐promoting regions, the TMN histaminergic neurons remain relatively more intact than orexinergic and nor‐adrenergic neurons. The preserved histaminergic neurons could be a potential target for addressing sleep dysfunction when neurons in the wake‐promoting system degenerate. We aimed to map neuropathological and molecular events in poorly understood histaminergic neurons across AD progression in the human TMN to help understand pathogenic features and guide therapeutic strategies.

**Method:**

We used unbiased stereology and double‐immunohistochemistry to quantify pTau (CP13) accumulation, the number of histaminergic, and total neurons in the TMN in subjects across progressive Braak stages (n = 20). Data were analyzed using the Wilcoxon signed‐rank test. We used a customized Neuropathology nCounter® (Nanostring) panel for proteomic analysis. Wald statistical test was used to compare the groups, and the genes were considered differentially expressed when the p‐value was <0.05.

**Result:**

TMN total neuronal count remained constant across Braak groups (BG), underscoring TMN’s resilience to AD compared to other wake‐promoting neurons. Histaminergic (HDC+CP13‐) neurons declined between BG 0‐2 and 5‐6 (p = 0.013). The number, proportion, and density of pTau inclusions in histaminergic and total TMN neurons increased across BG (p<0.05) (Fig. 1, Table 1). In Braak 5‐6 over 0‐2, we found 284 differentially expressed genes in TMN, of which 171 were upregulated. Gene ontology analysis demonstrated upregulation of cytokine‐cytokine receptor interaction pathways (p = 0.015). In Braak 5‐6 over 0‐2, histamine decarboxylase (HDC) expression was downregulated (lfc = ‐0.819, p = 0.37) with increased expression of histamine receptors HRH1 (lfc = 1.26, p = 0.028) and HRH2 (lfc = 1.76, p = 0.019) (Fig. 2).

**Conclusion:**

Stereological data revealed that the decline in histaminergic neurons is associated with pTau accumulation and reduced histamine synthesis rather than neuronal loss. This is corroborated by a decrease in HDC expression and an upregulation of histaminergic receptor expression (potentially compensatory) in late Braak stages. Interventions focused on pTau removal may succeed in reinstating histaminergic neuro‐transmission/‐modulation, improving cognition, and restoring sleep‐wake dysfunction in AD patients.